# Β3-Adrenergic Receptors and Prematurity-Related Diseases: A Systematic Review

**DOI:** 10.3390/children12121586

**Published:** 2025-11-22

**Authors:** Camilla Fazi, Carlo Dani

**Affiliations:** 1 Department of Neurosciences, Psychology, Drug Research and Child Health, University of Florence, 50139 Florence, Italy; camilla.fazi@unifi.it; 2 Division of Neonatology, Careggi University Hospital of Florence, 50134 Florence, Italy

**Keywords:** β3-ARs, prematurity, oxygen-related prematurity diseases, hypoxia/hyperoxia

## Abstract

**Background:** β3 adrenergic receptors (β3-ARs) have recently gained scientific attention due to their widespread body expression and their heterogeneous span of tissue-related functions. Recent research has hypothesized their involvement in the pathogenesis of some of the most common complications in preterm infants. The aim of the present systematic review is to examine the published scientific literature on the topic. **Methods:** PubMED/Medline and Cochrane databases were searched for studies reporting an association between β3-ARs, fetal development, and preterm newborns’ diseases. **Results:** Of 1596 articles found, 16 studies were selected for the review. Data currently available in the literature show that β3-ARs are upregulated in a hypoxic environment in several tissues and that their activation triggers a downstream cascade that promotes pro-angiogenic, anti-inflammatory, and immunoregulating effects, as well as metabolic adaptative processes and chemoresistance to xenobiotics. These characteristics seem to be central in the development of the fetus. **Conclusions:** Available preclinical data suggest the possible role of β3-ARs in the pathogenesis of a large number of premature newborn pathologies. Since fetal growth takes place in a low oxygenated environment, preterm delivery exposes newborns to a relatively hyperoxic setting while their development is not fully completed. Given the β3-ARs upregulation in a hypoxic environment, premature exposure to higher oxygen concentration levels affects their expression and their activity, probably derailing fetal normal development and injuring several organs. β3-ARs might therefore represent a central element in the pathogenesis of some of the main pathologies that preterm babies often develop.

## 1. Introduction

β-adrenoreceptors (β-ARs) are members of the 7-transmembrane receptor family, which belongs to the superfamily of G-protein-coupled receptors (GPCRs). They show a typical structure with three intra- and three extra-transmembrane loops with a glycosylated N-terminal extracellular domain and a cytosolic C-terminal tail, which contains phosphorylation sites for GPCR kinases [1].

β3-AR is the latest β-AR discovered: it was cloned for the first time in 1989 [2], and it was officially recognized as a member of the β-AR family in 1994 [3].

**Figure 1:**
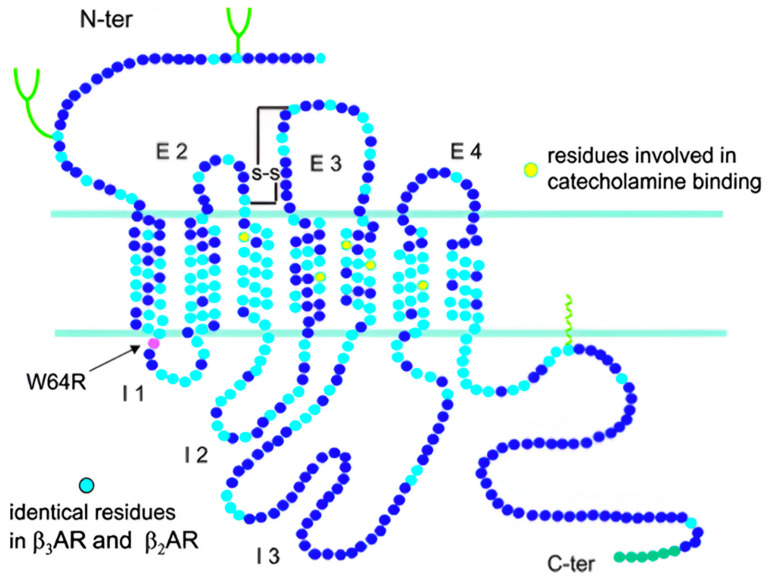
The beta 3 adrenoceptor structure. Source: http://www.biochemsoctrans.org/bst/035/0023/bst0350023.htm (accessed on 17 November 2025).

The gene encoding β3-AR, ADRB3, has been identified in several species, such as mouse, rat, sheep, bovine, goat, and dog [4]. In humans, ADRB3 is located on chromosome 8, and it shares 51% and 46% of sequence homology with the β1- and β2-AR amino-acid sequences, respectively [5]. It encodes a single 408–amino acid residue–long peptide chain receptor [6], which, unlike β1- and β2-ARs, lacks phosphorylation sites in the third intracellular loop and C-terminal tail: this characteristic makes these receptors quite resistant to agonist-induced desensitization, making them potentially suitable candidates for chronic treatments (Figure 1). However, this issue is still debated in the literature [7,8].

β3-AR expression has been reported in several tissues such as myocardium, retina, myometrium, adipose tissue, gallbladder, brain, urinary bladder, and blood vessels’ endothelium. It performs heterogeneous tissue-dependent functions including lipolysis and thermogenesis in adipocytes, vasodilation at endothelial level, negative cardiac inotropic effects, and relaxation of the smooth muscle cells of the detrusor in the bladder [6]. Nowadays, some relatively selective agonists and antagonists of β3-ARs are available: the first-generation agonists include BRL37344 and CL316243, the second-generation agonists include FDA-approved molecules like mirabegron (YM178), and others discontinued or under validation molecules such as amibegron (SR58611A), solabegron (GW427353), and ritobegron (KUC-7483) [6]. For the antagonists, two of the most widely used β3-AR antagonists are SR59230A and L748337 [6].

Although β3-AR antagonists’ expression seems to be limited in the tissues of adult healthy humans and rodents, in certain diseases, particularly hypoxia and hypoperfusion-driven pathologies, their expression increases, suggesting that hypoxia might promote their upregulation. This last feature has recently been thoroughly studied, particularly in the oncological and neonatological field [9].

The recent literature has, in fact, suggested that the developing fetus shares an uncanny number of environmental and adaptative strategies with tumoral cells, mainly mediated by β3-AR activation [10]. In brief, both fetal and tumoral cells start their development in a hypoxic, acidotic, and initially hypovascularized environment [11], requiring a pro-angiogenic response and several metabolic adaptations, such as the metabolic shift towards glycolysis even in the presence of oxygen, the so-called Warburg effect [12], which, through an increased export of lactate, facilitates the trophoblast or tumor infiltration [13]. Moreover, being *non-self*, they both locally “turn down” the immune system response, and they acquire resistance to xenobiotics [9]. A relevant number of studies performed both in vitro and in vivo on various typologies of tumors (melanoma, thyroid papillary carcinoma, T-cell lymphoma, neuroblastoma, etc.), demonstrated the increased level of β3-ARs and their involvement in the previously described adaptative mechanisms, as well as the beneficial effect of selective β3-AR antagonism [9]. β3-AR, for instance, promotes the tissutal pro-angiogenetic/vasculogenetic response once the Hypoxia Inducible Factor 1 (HIF-1) has been activated. HIF1, in hypoxic conditions, binds hypoxia-responsive elements (HREs) on DNA [14], activating the transcription of several hypoxia responsive genes including vascular endothelial growth factor (VEGF) [15], IL-6, IL-8, and FGF-2 [16]. The β3-AR blockade was found to prevent hypoxia-induced VEGF accumulation, and similarly, in mice bearing melanoma cells, β3-AR antagonism tackled tumor growth and vascularization [17], decreasing the NO production [18]. Moreover, studies performed with β-blockers and specific siRNA targeting also suggested β3-AR involvement in the abovementioned regulation of immune tolerance [19,20], in several metabolic adaptative mechanisms [13], and in the resistance to potentially toxic xenobiotics [9].

Considering the previously mentioned analogies with tumoral cells and the downstream pathways of β3-ARs, it has been hypothesized that fetal growth might also be related to β3-AR activation. Whether, in fact, the intrauterine environment is relatively hypoxic (2–8% of oxygen tension) [21], at birth. newborns are suddenly exposed to a significantly more oxygenated atmosphere, and in preterm newborns. this event happens when the vascularization and maturational processes of the baby are still incomplete. The lack of a trigger for β3-AR upregulation, previously provided by hypoxia, might therefore be the underlying cause of the failure of vascular progression, which is involved in most of the diseases related to prematurity. The eventual involvement of β3-ARs might, therefore, provide a brand-new interpretation of prematurity-related diseases which, despite the organ involved (brain, eye, lung, intestine, etc.), all share preterm exposure to a relatively hyperoxic environment (room air or supplemental oxygen therapy, often used in clinical practice to treat neonatal respiratory disorders) when the organs are not fully matured yet.

The objective of this systematic review is therefore to analyze the literature published on the involvement of β3-ARs in fetal development and in prematurity-related diseases.

## 2. Material and Methods

The present study was conducted and documented in accordance with the Preferred Reporting Items for Systematic Reviews and Meta-Analysis (PRISMA).

### 2.1. Literature Search and Screening Strategy

A systematic review was carried out to summarize the available results on the involvement of β3-ARs in fetal development and in prematurity-related diseases. To identify the eligible papers, we performed a systematic literature search on the PubMED and Cochrane databases.

In the current study, two well-trained authors independently performed a systematic literature search and scanned the outcomes of the search using a predetermined list of variables of interest.

The database search on PubMed was performed using the following string:

(“Receptors, Adrenergic, beta-3”[Mesh] OR “Adrenergic beta-3 Receptor Agonists”[Mesh] OR mirabegron OR “Adrenergic beta-3 Receptor Antagonists”[Mesh] OR ((Adrenergic Receptors OR adrenoreceptor OR adrenoceptor OR agonist OR stimulant OR stimulat*[tiab] OR antagonist OR blocking agent OR blocker) AND (beta-3*[tiab] OR beta3*[tiab] OR “beta 3*”[tiab] OR β3*[tiab])) OR “Receptors, Adrenergic, beta”[Mesh] OR β-adrenoceptor*[tiab] OR β-adrenoreceptor*[tiab] OR “beta adrenoceptor*”[tiab] OR “beta adrenoreceptor*”[tiab] OR “Adrenergic beta Receptor*”[tiab] OR “beta-Adrenergic Receptor*”[tiab]) NOT (TGF*[tiab] OR “transforming growth factor*”[tiab]) AND (“Infant, Premature”[Mesh] OR “Premature Birth”[Mesh] OR prematur*[tiab] OR preterm*[tiab] OR pre-term*[tiab] OR pre-matur*[tiab] OR “Fetal Development”[Mesh] OR “Fetal Organ Maturity”[Mesh] OR “Fetal Hypoxia”[Mesh] OR embryo*[tiab] OR “Embryonic Stem Cells”[Mesh] OR ((fetal[tiab] OR foetal[tiab] OR fetus*[tiab] OR foetus*[tiab] OR birth*[tiab]) AND (development*[tiab] OR growth*[tiab] OR growing[tiab] OR programming*[tiab] OR “organ maturity”[tiab] OR “functional maturity”[tiab] OR hypox*[tiab] OR anox*[tiab])) OR ((uterus OR endometrium OR pregnancy) AND hypox*[tiab]) OR white matter injury OR “White Matter/pathology”[Mesh] OR Necrotizing Enterocolitis OR bronchopulmonary dysplasia OR “broncho-pulmonary dysplasia” OR “lung dysplasia” OR Retinopathy of Prematurity OR oxygen-induced retinopathy OR ocular disease OR ((Retrolental OR retrolenticular) AND (fibroplasia OR dysplasia OR fibroplasia OR fibrosis)) OR Patent Ductus Arteriosus OR ((paten*[tiab] OR open[tiab] OR persisten*[tiab]) AND duct*[tiab] AND (arterios*[tiab] OR botalli*[tiab])))

This string was subsequently adapted for Cochrane research.

Moreover, to identify any article that may have been missed during the literature search, the bibliographies of the candidate articles were carefully checked.

### 2.2. Eligibility Criteria and Data Extraction

All articles were recovered and selected on the basis of the presence/absence of the search criteria. We imposed no types of publication restrictions on the search. No limit about the year of publication was set, and the final search was updated to December 2024.

First of all, the results obtained by the strings were analyzed, and duplicates were eliminated. As the first step of screening, through the analysis of the title and the abstract, we included all the articles about β3-ARs and all the publications in which, without specifying the subclass of β-ARs, pathologies or situations of interest (e.g., Fetal development, prematurity etc.) were analyzed. Concomitantly, all the articles in which the word “premature” was not associated with neonatal prematurity (e.g., premature senescence, premature pubarche, etc.) were excluded, as well as all the articles which explicitly analyzed only β1 and β2 ARs and those regarding agonists and antagonists without explicit reference to β3-ARs. Moreover, we excluded the generic literature on adrenergic receptors, all the works in which β3 was not related to the class of adrenergic receptors (e.g., TGF β3, Integrin β3, nrg-1 beta3, etc., Wnt/β3 catenin, etc.), and those in which β3-ARs were not related to pediatric pathologies. After this first screening, the articles selected were fully read, and only the articles specifically studying the relationship between β3 ARs and preterm pathologies were selected. In fact, articles which did not explicitly evaluate β3-ARs were excluded, as well as those which, although studying β3-ARs, analyzed pathologies not relevant to the review (e.g., gynecological diseases, oncological diseases, adulthood cardiovascular diseases, etc.). Moreover, the literature that only described specific characteristics of β3-Ars, such as their biochemical characteristics, were excluded. Likewise, articles about β3-ARs agonists and antagonists, which were limited to their pharmacokinetic characteristics or which studied pathologies outside our range of interest, were not selected. Finally, we excluded the articles which, despite the English title and abstract, were written in other languages.

During all those stages, two authors screened all of the titles, abstracts, and subsequently, the full-texts of the identified results. Between-reviewer inconsistencies concerning eligibility, inclusion, or data extraction were settled through discussion after reading the full text followed by mutual consensus. We were thus able to determine the final list of the included studies.

## 3. Results

### 3.1. Identification and Selection of Studies

**Figure 2:**
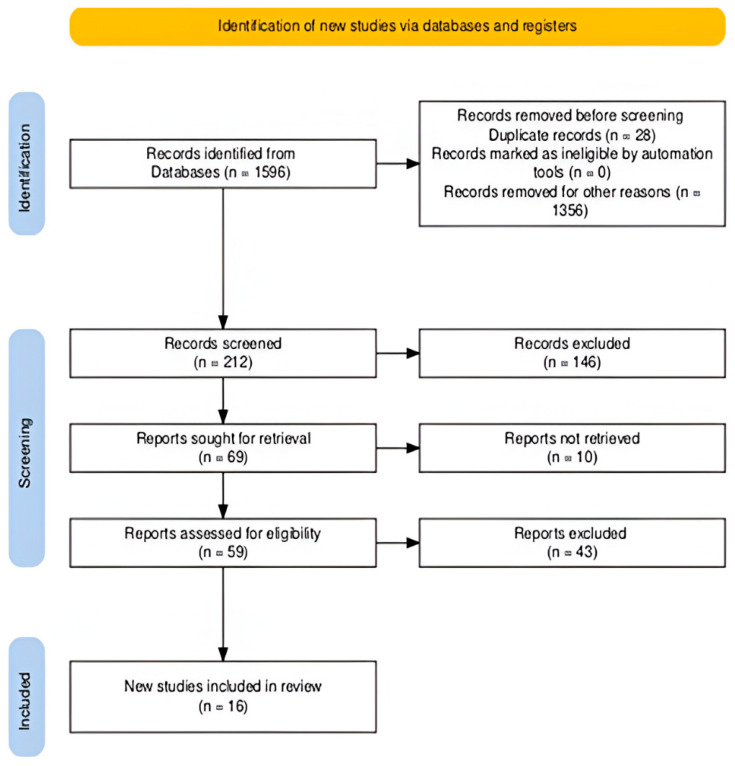
Algorithm of evaluated and selected studies.

A total of 1596 papers emerged, out of which, 28 were removed, as they were duplicates. From this collection, after having read the title and the abstract and having applied the previously described inclusion/exclusion criteria, 69 papers were selected. Out of those, 10 articles were excluded since the full text was not found even after using the necessary tools and resources. In total, 59 records were left to be retrieved for further screening. On reviewing full-text articles, three papers were excluded, because they were not in English, and of the remaining articles, 16 studies were finally included. The proposed algorithm with the number of studies is illustrated in Figure 2, and the articles finally selected are listed in Table 1.

### 3.2. Summary of Findings

Regarding the topic of study, namely the involvement of β3-ARs in fetal development and in preterm pathologies, nowadays, only preclinical studies are available in the scientific literature, the majority of which have been performed on murine models. One of the preterm pathologies in which β3-ARs involvement has been thoroughly studied is the Retinopathy of Prematurity (ROP). ROP is a multifactorial, evolutive, and potentially blinding disease, which affects premature newborns [31]. It is well-known that retinal vascularization is not completed until the last phases of gestation; therefore, the exposure to a relatively hyperoxic environment, which downregulates the expression of pro-angiogenic factors, might be deleterious in a preterm baby. This event, in fact, impairs the ongoing retinal vascularization, inducing the so-called ischemic phase of the ROP [32]. Due to the increasing metabolic requirements of the retina, retinal tissues cannot sustain an ischemic condition for long; therefore, proangiogenic factors are again upregulated, but they induce an aberrant neovascularization of the retina [33], which leads to the so-called proliferative retinopathy. Nowadays, medical efforts are directed to prevent ROP development through a careful monitoring of the levels of oxygen delivered to the baby. A recent study performed by Shukla et al. in 2019 suggested a “biphasic strategy” with a lower SpO2 target for the first weeks of life, when oxygen usually induces retinal ischemia, and a target over 95% after the 34th week, when the decrease in angiogenic processes is desirable [34]. When ROP is established, two available treatments are laser photocoagulation or intravitreal injection of anti-VEGF drugs [14]. Although several preclinical and trial studies focus on the administration of propranolol, a β1 and β2-AR β-blocker, during the hypoxic phase of the ROP [35], the finding that β3-ARs were particularly expressed in more aggressive and less responsive to propranolol cases sheds light on β3-ARs involvement in ROP pathogenesis [36]. Nowadays, it is known that hypoxia promotes catecholamine production [37] and that β3-Ars’ affinity for noradrenaline is about seven-fold higher than that of β2-ARs [38]. It has also been demonstrated that β3-ARs expression in the retina is upregulated in hypoxic environment and that β3-ARs colocalize with engorged retinal tufts [24], suggesting their sympathetic regulation of the angiogenic response to hypoxia. Moreover, it has also been shown that HIF-1 directly binds to the β3-AR enhancer region upregulating β3-ARs at transcriptional level and promoting endothelial cells’ migration and proliferation [27]. Additionally, several in vivo experiments confirmed the direct role of β3-ARs in regulating the production of proangiogenic factors in the hypoxic retina: in β1/β2-AR knockout mice with oxygen induced retinopathy (OIR), if β3-ARs were activated, retinal angiogenesis was more potently induced than in wild-type mice [39]. Furthermore, in retinal explants, hypoxia-induced upregulation of VEGF was blunted by β3-AR blockade or silencing, while it was increased by β3-AR agonism [40].

Concerning the heart, studies on cardiovascular tissue showed that, although β3-ARs are not particularly expressed in physiological conditions, they are upregulated in some pathological conditions such as heart failure, sepsis, and diabetes. Moreover, β3-AR stimulation in those experimental models exerts a cardioprotective protective effect [1]. For the field of neonatology, a pathology in which the role of β3-ARs was studied with regard to the cardiovascular system is the Patent Ductus Arteriosus (PDA). Ductus arteriosus (DA) is an extracardiac fetal shunt that connects the main pulmonary trunk to the descending aorta, and in utero, it allows the oxygenated blood to bypass the lungs, which are physiologically collapsed [41]. The main factors that maintain DA patent during intrauterine life are the low partial pressure of oxygen (pO2), the high levels of the endogenous vasodilators prostaglandin E2 (PGE_2_) circulating in the bloodstream, and the local production of NO [42]. In term infants, after delivery, the rapid increase in pO2, the decreased flow through the ductus arteriosus, and the reduction in vasodilators such as PGE_2_ induce the functional closure of the DA within 24–72 h [43], whereas the anatomic closure is complete within two to three weeks. In preterm infants, the DA does not usually spontaneously close, configuring the pathology of the PDA, which can require pharmacological or surgical treatment. In a recent article [44], studying the upregulation of β3-ARs in a hypoxic environment and their downstream vasodilatation through the NO pathway, evaluated the involvement of β3-ARs in the maintenance of DA patency in intrauterine life and in the pathogenesis of PDA. It was demonstrated that β3-ARs were highly expressed in mouse DA during intrauterine life, whereas they were less expressed in DA harvested from newborns early after delivery. In addition, acute or chronic administration of β3-AR antagonists in pregnant dams induced DA constriction and increased the wall thickness, supporting the hypothesis that β3-ARs are involved in the maintenance of DA patency.

No studies regarding β3-Ars’ direct or indirect involvement in the pathogenesis of bronchopulmonary disease, white matter injury, or necrotizing enterocolitis have been found in the literature, nor have studies been found on other neonatal pathologies such as icterus, glycemic regulation, hemostasis, etc. Of note, regarding the intestine, although not specifically addressing the problematic of necrotizing enterocolitis, in 2023, two papers shed light on the apparent protective role of β3-ARs in colonic and ileal alterations induced by hyperoxia [23,31]. Concerning the proximal colon, it has been demonstrated that hyperoxia, similar to the DA, reduces the expression of β3-ARs and, at the same time, induces histological alterations of the colonic structures, shortens the colonic length, and derails the normal development of the colonic myenteric plexus. The administration of BRL3734, a β3 specific agonist, has been able to prevent that phenomenon. Similarly, hyperoxia also damages the ileal mucosa and submucosa, impairs its vascularization, and produces a remarkable downregulation of β3-ARs expression; these effects seem to be counteracted by BRL37344, which also prevents the increase in specific markers of oxidative stress, therefore reducing local inflammation.

## 4. Discussion

**Figure 3:**
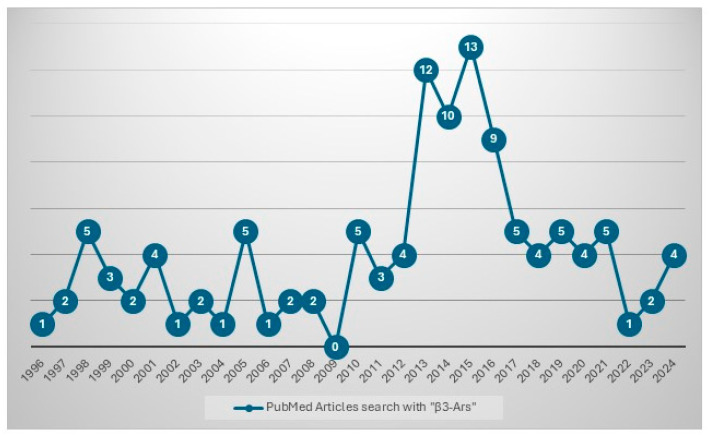
PubMed article search with “β3-ARs”.

In the last few years, the interest regarding β3-ARs has remarkably increased: just researching on Pubmed the key words “β3-ARs”, for example, we can remark that out of the 103 results published between 1996 and 2024, 53% of the articles have been published in the last 10 years (Figure 3).

From the systematic review of the literature performed and the wider study carried out for this article, it emerged that, to date, quite detailed information regarding β3 adrenergic receptors are available. In fact, even if “relatively new”, the horizons of the topic have rapidly expanded, and a large amount of information regarding animal and human tissue localization, gene and protein characteristics, biochemistry, some of the downstream biochemical pathways, up- and downregulation mechanisms, and possible selective agonists and antagonists have been described.

Although certainly still not exhaustive, this new knowledge can suggest a hypothesis regarding its clinical application, especially considering the evidence that β3-ARs tend to upregulate under hypoxia and to downregulate in a normoxic/hyperoxic environment. Concerning the field of neonatology, nowadays, there are no studies on human on animal models that demonstrate the direct and exclusive role of β3-ARs in any specific pathology and, therefore, the treatment or prevention with a β3-ARs agonist or antagonist of any particular disease. However, since the etiopathogenesis of most of those pathologies is multifactorial, this result was not likely to be expected, and on the contrary, it was somehow foreseeable. Nevertheless, the scientific literature provides us with several preclinical studies that give promising results, paving the way for future possible applications of β3-ARs in clinical research and maybe one day in neonatological practice.

Retracing the pathologies analyzed in the Results section, β3-adrenergic receptors (β3-ARs) seem to play a pivotal role in the regulation of vascular homeostasis within the developing retina, opening new perspectives in terms of the prevention and treatment of ROP. Their expression appears to increase under hypoxic conditions, amplifying the angiogenic response to oxygen deprivation. This, on one hand, supports physiological vascular growth in utero but, on the other, potentially contributes to pathological neovascularization when the oxygen balance is disturbed after preterm birth.

From a mechanistic perspective, the available evidence suggests that β3-AR activation might be beneficial in the early ischemic phase of ROP, as it could mitigate oxygen-induced vascular regression and preserve normal vascular maturation. Conversely, during the later proliferative phase, persistent β3-AR stimulation might sustain excessive VEGF-mediated angiogenesis, thus worsening disease severity. These observations, mostly derived from murine models, indicate a biphasic role for β3-ARs in retinal angiogenesis, where both the timing and degree of receptor modulation could critically determine the therapeutic outcomes [14].

Translationally, this highlights the potential, but also the complexity, of pharmacological β3-AR manipulation in ROP. β3-AR agonists could theoretically support vascular stability in the early stages, whereas antagonists might suppress aberrant proliferation later on. Although no β3-selective antagonists are yet clinically available, the existence of FDA-approved β3 agonists (e.g., mirabegron, vibegron) opens a plausible route for experimental repurposing. Further studies should clarify the safety and temporal dynamics of such interventions in the fragile preterm population.

β3-adrenergic receptors (β3-ARs) have also been investigated in relation to the pathogenesis of patent ductus arteriosus (PDA). The main factors that maintain ductus arteriosus (DA) patency during intrauterine life are the low partial pressure of oxygen (pO_2_), the high circulating levels of the endogenous vasodilator prostaglandin E_2_ (PGE_2_), and the local production of nitric oxide (NO) [42]. In preterm infants, the mechanisms that normally lead to DA closure within 24–72 h after birth are often insufficient, and the DA may remain patent, potentially causing medical issues that might require pharmacological or surgical treatment. Experimental studies in murine models have shown that β3-ARs are highly expressed in the hypoxic intrauterine environment, that their expression decreases after birth, and that they may take part in the modulation of DA patency [44].

From a mechanistic perspective, β3-AR activation seems to be able to promote smooth-muscle relaxation, whereas its antagonism could contribute to DA constriction and an increase in the wall thickness.

Translationally, this dual action opens the possibility of manipulating β3-ARs both prenatally and postnatally: agonists could be explored as potential therapies for fetal intrauterine DA constriction, whereas antagonists might enhance postnatal ductal closure in refractory PDA cases. Although these applications remain theoretical, they provide a rationale for further studies investigating β3-AR modulation as a potential adjunct in PDA management.

Within the gastrointestinal system, β3-adrenergic receptors (β3-ARs) have recently attracted attention for their potential role in intestinal maturation and protection against oxygen-related injury. Although direct evidence linking β3-ARs to necrotizing enterocolitis (NEC) is not yet available, several studies have highlighted their involvement in the adaptive responses of the neonatal gut to oxygen exposure. Experimental data from hyperoxia-exposed murine models demonstrated that β3-AR expression is markedly reduced in both the proximal colon and ileum under high oxygen conditions, in parallel with histological alterations, a shortened colonic length, impaired vascularization, and disrupted development of the myenteric plexus. Administration of BRL37344, a selective β3-AR agonist, was able to tackle these changes, suggesting that β3-AR signaling contributes to maintaining the structural and vascular integrity of the developing intestine. As observed for the retina and the ductus arteriosus, colonic and ileal tissues also display a biphasic pattern of β3-AR expression in relation to the oxygen levels. This has led to hypotheses regarding their physiological role in intestinal development: β3-ARs may promote morphogenesis during intrauterine life, while their postnatal downregulation could favor the completion of the maturation process [21]. If this theory is confirmed, β3-AR agonists might enhance organ vascularization and maturation, thereby helping to prevent intestinal diseases such as NEC, in which premature exposure to a hyperoxic environment disrupts vascularization and normal tissue development, increasing the risk of hypoxic-ischemic injury [29].

From a mechanistic perspective, β3-AR activation appears to exert a protective role, potentially preserving mucosal perfusion and neuronal development under hyperoxic conditions.

Translationally, this supports the hypothesis that β3-AR agonists could enhance intestinal maturation and resilience, reducing susceptibility to ischemic and inflammatory injury in preterm infants. Further studies are warranted to confirm these effects and to clarify whether β3-AR–mediated pathways may represent a therapeutic target for the prevention of NEC and other gastrointestinal complications of prematurity.

No studies directly addressing the role of β3-adrenergic receptors (β3-ARs) in neonatal white matter injury or other brain lesions were identified in the present review. However, given the evidence from other organs that β3-ARs are upregulated under hypoxic conditions and can modulate oxidative stress and vascular tone, it is plausible that similar mechanisms might operate in the developing brain. Further research is warranted to explore whether β3-AR–mediated pathways could contribute to neurovascular protection in preterm infants.

At present, no β3-AR–targeted therapies have been specifically developed or approved for neonatal use. However, the existence of FDA-approved β3-AR agonists such as mirabegron and vibegron, currently indicated for the treatment of overactive bladder in adults, opens intriguing perspectives for potential pharmacological repurposing. Both compounds have demonstrated favorable safety profiles in adult and pediatric populations for non-neonatal indications, and their mechanisms of action, mainly involving β3-AR–mediated vasodilation, metabolic modulation, and anti-inflammatory effects, are consistent with the pathways implicated in several complications of prematurity. While their direct application in neonates remains purely theoretical, these agents provide a valuable proof of concept that β3-ARs can be safely and selectively modulated in humans. Future translational studies should therefore explore whether such drugs, or next-generation β3-AR modulators with improved selectivity and pharmacokinetics, might offer novel strategies to prevent or mitigate oxygen- and hypoxia-related pathologies in preterm infants.

## 5. Conclusions

Pre-clinical studies suggest that β3-ARs are an emerging novel promising therapeutic target for conditions in whose pathogenesis hypoxic-ischemic events are involved. If these findings are confirmed, the development of drugs acting on β3-ARs could contribute to the prevention and/or treatment of severe complications of prematurity such as NEC, ROP, and PDA.

Moreover, since it is plausible that the same mechanisms of immaturity and impaired development underlying these conditions are also involved in other neonatal diseases typical of preterm infants, such as bronchopulmonary dysplasia and white matter injury, further studies in this field would be highly advisable and valuable, as they could pave the way for new therapeutic approaches to conditions for which prevention is challenging, and treatment options remain limited.

## Figures and Tables

**Table 1:** List of selected articles.

Nr	Ref.	Authors	Title	Journal, Date, Issue, Page	doi
1	[22]	Casteilla L, Muzzin P, Revelli JP, Ricquier D, Giacobino JP	Expression of beta 1- and beta 3-adrenergic-receptor messages and adenylate cyclase beta-adrenergic response in bovine perirenal adipose tissue during its transformation from brown into white fat	Biochem J. 1994 Jan 1;297 (Pt 1)(Pt 1):93–7	10.1042/bj2970093
2	[23]	Wang X, Cui Y, Tong X, Ye H, Li S	Effects of the Trp64Arg polymorphism in the beta3-adrenergic receptor gene on insulin sensitivity in small for gestational age neonates	J Clin Endocrinol Metab. 2004 Oct;89(10):4981–5.	10.1210/jc.2003-032027
3	[24]	Ristori C, Filippi L, Dal Monte M, Martini D, Cammalleri M, Fortunato P, la Marca G, Fiorini P, Bagnoli P	Role of the adrenergic system in a mouse model of oxygen-induced retinopathy: antiangiogenic effects of beta-adrenoreceptor blockade	Invest Ophthalmol Vis Sci. 2011 Jan 5;52(1):155–70	10.1167/iovs.10-5536
4	[25]	Cavallaro G, Filippi L, Bagnoli P, La Marca G, Cristofori G, Raffaeli G, Padrini L, Araimo G, Fumagalli M, Groppo M, Dal Monte M, Osnaghi S, Fiorini P, Mosca F	The pathophysiology of retinopathy of prematurity: an update of previous and recent knowledge	Acta Ophthalmol. 2014 Feb;92(1):2–20	10.1111/aos.12049
5	[13]	Schena G, Caplan MJ	Everything You Always Wanted to Know about β_3_-AR * (* But Were Afraid to Ask)	Cells. 2019 Apr 16;8(4):357	10.3390/cells8040357
6	[26]	Calvani M, Dabraio A, Subbiani A, Buonvicino D, De Gregorio V, Ciullini Mannurita S, Pini A, Nardini P, Favre C, Filippi L	β3-Adrenoceptors as Putative Regulator of Immune Tolerance in Cancer and Pregnancy	Front Immunol. 2020 Sep 2;11:2098	10.3389/fimmu.2020.02098
7	[22]	Pini A, Fazi C, Nardini P, Calvani M, Fabbri S, Guerrini A, Forni G, La Marca G, Rosa AC, Filippi L	Effect of Beta 3 Adrenoreceptor Modulation on Patency of the Ductus Arteriosus	Cells. 2020 Dec 7;9(12):2625	10.3390/cells9122625
8	[14]	Filippi L, Cammalleri M, Amato R, Ciantelli M, Pini A, Bagnoli P, Dal Monte M	Decoupling Oxygen Tension From Retinal Vascularization as a New Perspective for Management of Retinopathy of Prematurity. New Opportunities From β-adrenoceptors	Front Pharmacol. 2022 Jan 21;13:835771	10.3389/fphar.2022.835771
9	[27]	Amato R, Pisani F, Laudadio E, Cammalleri M, Lucchesi M, Marracci S, Filippi L, Galeazzi R, Svelto M, Dal Monte M, Bagnoli P	HIF-1-Dependent Induction of β3 Adrenoceptor: Evidence from the Mouse Retina	Cells. 2022 Apr 8;11(8):1271	10.3390/cells11081271
10	[13]	Filippi L, Pini A, Cammalleri M, Bagnoli P, Dal Monte M	β3-Adrenoceptor, a novel player in the round-trip from neonatal diseases to cancer: Suggestive clues from embryo	Med Res Rev. 2022 May;42(3):1179–1201	10.1002/med.21874
11	[21]	Filippi L, Nardini P, Zizi V, Molino M, Fazi C, Calvani M, Carrozzo F, Cavallaro G, Giuseppetti G, Calosi L, Crociani O, Pini A	β3 Adrenoceptor Agonism Prevents Hyperoxia-Induced Colonic Alterations	Biomolecules. 2023 Dec 6;13(12):1755	10.3390/biom13121755
12	[15]	Cammalleri M, Amato R, Dal Monte M, Filippi L, Bagnoli P	The β3 adrenoceptor in proliferative retinopathies: “Cinderella” steps out of its family shadow	Pharmacol Res. 2023 Apr;190:106713.	10.1016/j.phrs.2023.106713
13	[4]	Pasha A, Tondo A, Favre C, Calvani M	Inside the Biology of the β3-Adrenoceptor	Biomolecules. 2024 Jan 29;14(2):159	10.3390/biom14020159
14	[28]	Scaramuzzo RT, Crucitta S, Del Re M, Cammalleri M, Bagnoli P, Dal Monte M, Pini A, Filippi L	β3-adREnoceptor Analysis in CORD Blood of Neonates (β3 RECORD): Study Protocol of a Pilot Clinical Investigation	Life (Basel). 2024 Jun 19;14(6):776	10.3390/life14060776
15	[29]	Nardini P, Zizi V, Molino M, Fazi C, Calvani M, Carrozzo F, Giuseppetti G, Calosi L, Guasti D, Biagini D, Di Francesco F, Filippi L, Pini A	Protective Effects of Beta-3 Adrenoceptor Agonism on Mucosal Integrity in Hyperoxia-Induced Ileal Alterations.	Antioxidants (Basel). 2024 Jul 18;13(7):863.	10.3390/antiox13070863
16	[30]	Melecchi A, Canovai A, Amato R, Dal Monte M, Filippi L, Bagnoli P, Cammalleri M.	Agonism of β3-Adrenoceptors Inhibits Pathological Retinal Angiogenesis in the Model of Oxygen-Induced Retinopathy	Invest Ophthalmol Vis Sci. 2024 Aug 1;65(10):34	10.1167/iovs.65.10.3

## Data Availability

The data that support the findings of this study are available in PubMed and the Cochrane library at the public domain https://pubmed.ncbi.nlm.nih.gov/, accessed on 17 November 2025, and https://www.cochranelibrary.com/, accessed on 17 November 2025.
